# TanP: A Multifunctional Anionic Peptide From *Tityus stigmurus* Scorpion Venom

**DOI:** 10.3389/fmolb.2021.785316

**Published:** 2022-01-17

**Authors:** Menilla Maria Alves de Melo, Verônica da Silva Oliveira, Moacir Fernandes de Queiroz Neto, Weslley de Souza Paiva, Manoela Torres-Rêgo, Sérgio Ruschi Bergamachi Silva, Daniel de Lima Pontes, Hugo Alexandre Oliveira Rocha, Miguel Ângelo Fonseca de Souza, Arnóbio Antônio da Silva-Júnior, Matheus de Freitas Fernandes-Pedrosa

**Affiliations:** ^1^ Laboratory of Pharmaceutical Technology and Biotechnology, Department of Pharmacy, Federal University of Rio Grande do Norte, Natal, Brazil; ^2^ Laboratory of Coordination Chemistry and Polymers, Institute of Chemistry, Federal University of Rio Grande do Norte, Natal, Brazil; ^3^ Laboratory of Natural Polymer Biotechnology, Department of Biochemistry, Federal University of Rio Grande do Norte, Natal, Brazil; ^4^ Laboratory of Synthesis and Isolation of Organic Compounds, Chemistry Institute, Federal University of Rio Grande do Norte, Natal, Brazil; ^5^ Institute of Brain, Federal University of Rio Grande do Norte, Natal, Brazil; ^6^ Laboratory of Computational Chemistry, Institute of Chemistry, Federal University of Rio Grande do Norte, Natal, Brazil

**Keywords:** anionic peptide, chelating, antioxidant, immunomodulatory, *Tityus stigmurus* scorpion

## Abstract

Anionic peptides of scorpions are molecules rich in aspartic and/or glutamic acid residues and correspond to a class of peptides without disulfide bonds that are still little explored. TanP is a linear anionic peptide (50 amino acid residues and net charge −20) present in the venom gland of the scorpion, *Tityus stigmurus*, with chelating properties for Cu^2+^ ion and immunomodulatory properties. The therapeutic application of chelating molecules is related to cases of acute or chronic intoxication by metals, neurodegenerative diseases, hematological diseases, healing of skin wounds, cardiovascular diseases, and cancer. In this approach, the chelating activity of TanP was evaluated in relation to new metal ions (Fe^2+^ and Zn^2+^) of biological importance, as well as its antioxidant, hemostatic, immunomodulatory, and healing potential, aiming to expand the biological and biotechnological potential of this peptide. TanP (25 µM) was able to form stable complexes with Fe^2+^ in a ratio of 1:5 (TanP: Fe^2+^). Theoretical results suggest that TanP can work as a sensor to identify and quantify Fe^2+^ ions. The fluorescence intensity of TanP (1.12 µM) decreased significantly after the addition of Fe^2+^, obtaining the highest ratio 1: 7.4 (TanP: Fe^2+^) that led to the lowest fluorescence intensity. For Zn^2+^, no relevant spectral change was noted. TanP (50 µM) showed a maximum of 3% of hemolytic activity, demonstrating biocompatibility, as well as exhibiting a 1,1-diphenyl-2-picrylhydrazyl radical–scavenging activity of above 70% at all the concentrations tested (1–25 μM), and 89.7% iron-chelating activity at 25 μM and 96% hydroxyl radical–scavenging activity at 73.6 μM. In addition, TanP (12.5 and 25 µM) revealed an anticoagulant effect, prolonging the clotting time in prothrombin time and activated partial thromboplastin time assays, with no fibrinogenolytic activity. TanP (12.5 and 25 µM) induced the release of TNF-α by murine macrophages, in the absence of lipopolysaccharides, with a concentration-dependent increase and also stimulated the migration of 3T3 cells in the *in vitro* healing assay. Thus, TanP revealed a multifunctional potential, being useful as a prototype for the development of new therapeutic and biotechnological agents.

## Introduction

Anionic, or acidic, peptides are a new class of scorpion venom peptides, which have been rarely identified and poorly characterized so far but are widely present in the venom glands of all detected species of scorpions (Shi et al., 2018). These molecules are peptides without disulfide bonds, rich in aspartic and glutamic acid residues, with an isoelectric point less than 5.0, showing high hydrophilic properties and secondary structures composed by random regions, α-helix domains, and spiral structures (Nie et al., 2012; Shi et al., 2018).

Regarding *Tityus* genus, an abundant presence of anionic peptides has been reported, with the observation that 27.22, 7.75, 4.9, and 3.14% of the total toxins expressed in the venom of scorpions *Tityus stigmurus* (Almeida et al., 2012), *Tityus serrulatus* (Alvarenga et al., 2012), *Tityus bahiensis* (Oliveira et al., 2015), and *Tityus obscurus* (de Oliveira et al., 2018), respectively, correspond to anionic peptides. The scorpions *Centruroides tecomanus* (Valdez-Velázquez et al., 2013), *Buthus martensii* Karsch (Zeng et al., 2005), *Centruroides hirsuti*-*palpus* (Valdez-Velázquez et al., 2020), and *Mesobuthus eupeus* (Baradaran et al., 2018) also showed an anionic peptide in the venom composition.

In a previous study carried out by our research group, an anionic peptide present in the *T. stigmurus* scorpion venom gland, named TanP (*T. stigmurus* anionic peptide) was characterized for the first time, with a negative charge of −20 and a theoretical isoelectric point of 2.75. *In vitro* assays have demonstrated that TanP has the chelating activity of Cu^2+^ ions and revealed an immunomodulatory potential, since it induced the proliferation of macrophages and reduced the release of nitric oxide by these cells, in the presence of lipopolysaccharides (LPS) (Melo et al., 2017).

Anionic peptides from vertebrate and invertebrate animals have demonstrated antimicrobial, immunomodulatory, and metal-chelating action (Lai et al., 2002; Silva et al., 2009; Segura-Ramírez and Silva Júnior, 2018; Zhang et al., 2021), which are part of the host defense system in scorpions and other phyla (Valdez-Velázquez et al., 2020). The use of metallopharmacology techniques (Flora and Pachauri, 2010) is useful for restoring the normal healthy physiology of the body, with wide therapeutic applications in cases of acute or chronic intoxication by metals (Andersen, 2004; Ward et al., 2012; Smith, 2013), neurodegenerative diseases (Ward et al., 2012; Ben-Shushan and Miller, 2021), hematological diseases (Pretorius et al., 2014; Leitch and Gattermann, 2019), cutaneous wound healing (Wright et al., 2014), cardiovascular diseases (Smith, 2013), and cancer (Yu et al., 2012; Antoniades et al., 2013). The use of small molecules or chelating peptides corresponds to an attractive strategy both to understand the fundamentals of biological regulation of the metal and to develop new therapies (Wang and Franz, 2016; Caetano-Silva et al., 2021; Zeng et al., 2021).

Recently, some researchers have demonstrated the multifunctionality of linear peptides present in scorpions (D’Suze et al., 2010; Gao et al., 2010; Guo et al., 2013; Ortiz et al., 2015; Daniele-Silva et al., 2016, 2021; Guilhelmelli et al., 2016; Ojeda et al., 2016; Cesa-Luna et al., 2019), and their antibacterial, antifungal, hypotensive, anticancer, and immunomodulatory activities have been identified (Zeng et al., 2012; Almaaytah et al., 2013; Daniele-Silva et al., 2016, 2021; Machado et al., 2016; Torres-Rêgo et al., 2019; Furtado et al., 2020). However, little researches about anionic peptides of these arachnids have been reported. In this approach, the evaluation of the chelating activity of TanP was carried out in relation to new metals of biological importance and expanding the investigation of its biological potential.

## Materials and Methods

### *Tityus stigmurus* Anionic Peptide (TanP) Synthesis

The peptide TanP was deduced from the cDNA clone TSTI0006C obtained from *T. stigmurus* transcriptome (Almeida et al., 2012). The synthetic mature peptide (YPASFDDDFDALDDLDDLDLDDLLDLEPADLVLLDMWANMMDSQDFEDFE) was obtained from Aminotech (Minas Gerais, Brazil) with purity higher than 90% and was maintained at −20°C until the time of use. The peptide tests were conducted under authorization from the National System for Management of Genetic Heritage and Associated Traditional Knowledge (SisGen), under the registration number AAF17D9 (April 24th, 2018).

### TanP—Bivalent Metal Reactivity Assay

The reactivity of TanP with bivalent ions (Fe^2+^ and Zn^2+^) was evaluated by spectroscopy technique, using the methodology described by Melo et al. (2017). In brief, TanP (25 μM), in the absence or presence of the ions (Fe^2+^ and Zn^2+^) at increasing concentrations (0–175 μM) was incubated at room temperature (25°C) for 5 min. Then, the absorbance of the mixture was measured between 190 and 800 nm in a quartz cuvette (1 cm optical path), using the spectrophotometer Agilent 8453 UV-visible Spectroscopy System (Agilent, Santa Clara, California, United States of America) with a DAD detector. Electronic spectra were generated for the evaluation of complexation. The stoichiometric ratio for the complex was obtained from the extrapolation calculation of the lines, equaling the values of the y-axis of each of the equations of the lines, thus obtaining the value of x, corresponding to the concentration of the metal in the sample.

### Fluorescence Emission Spectrophotometry

Fluorescence spectra were recorded with a spectrofluorometer (RF-5301PC model Shimadzu, Japan), with 280 nm excitation wavelength. The emission spectrum was recorded in the range of 300–500 nm. All measurements were performed with wide slits of excitation and emission equal to 1.5 and 15 nm, respectively. The TanP sample (1.12 µM) was titrated with the increasing concentrations of Fe^2+^ (0–47.7 µM) or Zn^2+^ (0–50 µM) ions, obtained from FeSO_4_·7H_2_O or ZnCl_2_ solutions, respectively. After 5 min of the addition of the ions, the fluorescence emission spectra were obtained, and the results were expressed as fluorescence intensity vs. wavelength (nm) (Croney, 2001; Caetano-Silva et al., 2017). All samples were measured in a quartz cuvette (path length 1 cm). For the titration of the iron and zinc ions, 12 and 14 spectra were obtained, respectively.

### Computational Methods

Theoretical calculations were focused on proposing a probable mechanism of interaction between the metallic center and TanP, particularly through their carboxylate groups belonging to the side chains of the aspartate and glutamate residues. Previous experimental results for Cu^2+^ ions and TanP indicate that this chelating process takes place via carboxylate pairs located throughout the tertiary structure (Melo et al., 2017). Hence, a complexation model, among an acetate pair, water molecules, and metal ions (Fe^2+^ and Zn^2+^), was tried to support and understand the chelating effect. The use of acetate to describe the Asp/metals interaction is feasible since the difference in local electron density is minimal and the interaction site remains the same. A conformational search was carried out using the Conformer–Rotamer Ensemble Sampling Tool CREST version 2.10.2 (Pracht et al., 2020) with the xtb program package (Bannwarth et al., 2021). The semiempirical tight-binding–based quantum chemistry method GFN2-xTB was used in the framework of meta-dynamics to globally explore the conformers (Grimme, 2019). The best conformer for each metal was subjected to density functional theory (DFT) geometry optimization at the BP86 (Perdew, 1986; Becke, 1988)/def2-TZVP (Perdew, 1986; Becke, 1988; Weigend and Ahlrichs, 2005) level of theory in an implicit solvent (water) using the polarizable continuum model (IEFPCM) (Scalmani and Frisch, 2010). After optimization calculations, the TD-DFT (Pracht et al., 2020) model for predicting vertical transition energies of the first excited state was performed at the CAM-B3LYP (Yanai et al., 2004)/def2-TZVP. Furthermore, natural bond orbital (NBO) (Foster and Weinhold, 1980; Reed and Weinhold, 1983) and natural transition orbital (NTO) (Martin, 2003) analyses were also carried out to provide a more intuitive picture of the molecular orbitals. The Gaussian 16 program was used for all DFT calculations (Frisch et al., 2016).

### Hemolytic Activity

The hemolytic effect of TanP was determined previously by de Menezes et al. (2014), with modifications. In brief, the suspension of 1% (v/v) healthy human erythrocytes (blood group O^+^ Rh^+^) was incubated with TanP (1.56–50 µM) for 1 h at 37°C. After this period, the samples were centrifuged at 1,500 rpm for 10 min at 25°C (Eppendorf^®^ 5424 R, Germany). Then, 200 μl of the supernatant was transferred to a 96-well microplate, and the absorbance of the hemoglobin was measured at 540 nm using the microplate reader (Epoch-Biotek^®^, Vermont, United States of America). Triton X-100 1% (v/v) and phosphate-buffered saline (137 mM NaCl, 3 mM KCl, 1.5 mM KH_2_PO_4_, and 10 mM Na_2_HPO_4_; pH, 7.4) were used as the positive control (100% hemolysis) and the negative control (0% hemolysis), respectively. The results were expressed as the percentage of red cell lysis compared with the positive control (100% lysis). For the use of blood from a healthy human donor, the project was previously approved by the research ethics committee of the Hospital Universitário Onofre Lopes—Huol/UFRN, under the number 3127063.

### *In Vitro* Antioxidant Activity

#### 1,1-Diphenyl-2-picrylhydrazyl Scavenging Assay

The 1,1-diphenyl-2-picrylhydrazyl (DPPH) radical–scavenging activity evaluated the ability of TanP to donate hydrogen or scavenge the DPPH radical in an ethanol solution. This method is based on the reduction of the DPPH radical (very unstable nitrogen radical and is purple). When reacting with reducing substances, that is, antioxidants, it is transformed into diphenyl-picryl-hydrazine (DPPH-H) which is yellow. The DPPH radical–scavenging effect was measured using the method described by Melo-Silveira et al. (2014), with modifications. In brief, 100 μl of TanP (1–25 µM) was mixed with 100 μl of ethanol solution of DPPH (150 µM) and incubated for 30 min, protected from light, at room temperature (25°C). After incubation, the absorbance was measured at 517 nm. The DPPH free-radical–scavenging activity (DPPH-FSA) was determined using the following equation, where the blank sample is ethanol solution and the blank control is DPPH solution:

$$\text{DPPH}-\text{FSA }\left(\text{\%}\right)=\left[1-\;\frac{\left(\text{absorbance of sample}-\text{absorbance of blank sample}\right)\;}{\left(\text{absorbance of control}-\text{ absorbance of blank control }\right)}\right]\text{X }100$$

#### Hydroxyl Radical–Scavenging Assay

The hydroxyl radical–scavenging effect of TanP was investigated using Fenton's reaction (Fe^2+^ + H_2_O_2_ → Fe^3+^ + OH^−^ + OH•) as previously described in the literature, with few modifications (Melo-Silveira et al., 2014). In 96-well microplates, TanP (1–73.6 µM) was incubated with the reagent solution [10 mM ferrous sulfate, 10 mM ethylenediaminetetraacetic acid (EDTA), 2 mM sodium salicylate, and 30% hydrogen peroxide in 150 mM sodium phosphate buffer; pH 7.4], at 37°C for 60 min, leading to the formation of the hydroxyl radical. Then, the absorbance of hydroxyl radicals was measured at 510 nm. Tubes in the absence of hydrogen peroxide were used as blank tubes. The results were expressed as percentage of scavenging compared to the standard gallic acid (0.25–2 mg/ml).

#### Superoxide Radical–Scavenging Assay

The superoxide radical–scavenging activity was determined as described by Melo-Silveira et al. (2014). The reaction mixture containing TanP at different concentrations (1–25 µM), 50 mM sodium phosphate buffer (pH 7.4), 65 mM methionine, 0.5 mM EDTA, 0.375 nitrotetrazolium blue chloride, and 0.5 mM riboflavin were exposed to 15-min illumination with a fluorescent lamp. The change in color was measured (560 nm) with a spectrophotometer. The control and blank mixtures were prepared. The blank was protected from light. The results were expressed as the percentage of hydroxyl radical–scavenging activity, as shown in the previous equation.

$$\text{\% radical scavenging }=\left[\;\frac{\left(\text{absorbance of control}-\text{absorbance of sample}\right)\;}{\left(\text{absorbance of control}-\text{ absorbance of blank }\right)}\right]\text{X }100$$

#### Iron-Chelating Assay

The ability to chelate iron ions was assessed as previously described in the literature, with modifications (Wang et al., 2008). The samples of TanP (1–25 µM) were added to the reaction solution (2 mM FeCl_2_ and 5 mM ferrozine) and incubated for 10 min at 37°C. The absorbance was measured at 562 nm. The chelating activity was expressed as the chelation percentage in relation to a blank (absence of sample). EDTA (0.1 mg/ml) was used as the positive control.

#### Copper Chelation Assay

The copper chelation test was performed as described previously (Presa et al., 2018). In brief, 96-well microplates with a reaction mixture containing different concentrations of TanP (1–25 µM), pyrocatechol violet (4 mM), and copper II sulfate pentahydrate (50 mg/ml) were homogenized with the aid of a micropipette, and the absorbance of the solution was measured at 632 nm using a microplate reader (SpectraMax^®^ M2/M2e, Molecular Devices, São José, California, United States of America).

#### Reducing Power Assay

The reducing power of the samples was examined according to Presa et al. (2018). In brief, different sample concentrations (1–25 µM) were added to a solution of 200 mM sodium phosphate buffer (pH 6.6) and potassium ferricyanide (10 mg/ml). After incubation in a water bath at 50°C for 20 min, trichloroacetic acid (10% w/v) and iron III chloride (0.1% w/v) were added. The mixture was stirred, and the absorbance (700 nm) was measured using a microplate reader. The results were expressed as the percentage of activity observed for 0.1 mg/ml (highest activity) ascorbic acid.

### Determination of Anticoagulant Activity

#### Prothrombin Time Test

The action of TanP on the extrinsic coagulation pathway was evaluated by prothrombin time (PT) test, as previously described in the literature, with modifications (Félix-Silva et al., 2014). The test was carried out using commercial reagent kits (CLOT Bios Diagnostica®, São Paulo, Brazil). The plasma (70 μl) was mixed with 30 μl of TanP (2, 12.5, and 25 μM) and incubated at 37°C for 5 min. Then, 200 μl of the PT assay reagent (rabbit brain extract and calcium chloride) pre-warmed at 37°C for 10 min was added, and the clotting time was recorded by a digital coagulometer (“Laser Sensor” Clotimer, CLOT, São Paulo, Brazil). Plasma alone (only with vehicle) was used as the control (in the absence of anticoagulant activity). The plasma with heparin (1 IU/ml) (Cristalia®, São Paulo, Brazil) was used as the positive control.

#### Activated Partial Thromboplastin Time Test

The action of TanP in intrinsic and common pathways of the coagulation cascade was evaluated by activated partial thromboplastin time (aPTT) assay, as previously described in the literature, with modifications (Félix-Silva et al., 2014). The test was carried out using commercial reagent kits (CLOT Bios Diagnostica). The plasma (70 μl) was mixed with 30 μl of TanP (2, 12.5, and 25 μM) and incubated at 37°C for 5 min. Then, 50 μl of the pre-warmed aPTT reagent (rabbit brain extract and ellagic acid) was added and incubated at 37°C for 3 min. After incubation, pre-warmed (37°C), 50 μl of 25 mM calcium chloride was added and the clotting time was recorded by a digital coagulometer (“Laser Sensor” Clotimer, CLOT). The plasma alone (only with the vehicle) was used as the control (in the absence of anticoagulant activity). The plasma with heparin (1 IU/ml) (Cristalia^®^) was used as the positive control.

#### Fibrinogenolytic Activity

The effect of TanP on fibrinogen was evaluated by sodium dodecyl sulfate–polyacrylamide gel (SDS-PAGE) electrophoresis (Félix-Silva et al., 2014). The separation conditions were: voltage of 130 V, amperage of 50 mA, and power of 90 W. The different concentrations of TanP (2–178 μM) were mixed with 50 μg of the fibrinogen (2 μg/μl) and then incubated for 240 min at 37°C. The reaction was stopped by adding 25 μl of sample buffer containing 10% β-mercaptoethanol and 2% SDS, followed by boiling for 5 min, and subjected to SDS-PAGE (12%). The fibrinogen-hydrolyzing pattern was visualized by staining with Coomassie brilliant blue R-250. Fibrinogen alone was used as the control, for visualization of the intact fibrinogen profile. The *Bothrops leucurus* venom (10 μg) was used as the positive control for the fibrinogenolytic activity. A sample containing only the peptide in the highest concentration (178 μM) was used for control. Subsequently, the gel was digitized and the image was binarized to measure the area of one of the bands referring to fibrinogen degradation, using ImageJ 1.44p software (National Institutes of Health, Maryland, United States of America).

### *In Vitro* Immunomodulatory Activity

Initially, TanP cytotoxicity by the 3-(4,5-dimethylthiazol-2-yl)-2,5-diphenyltetrazolium bromide (MTT) reduction method to RAW 264.7 cells had been evaluated. The immunomodulatory activity was evaluated by quantitation of IL-6 and TNF cytokine levels in the TanP-treated RAW 264.7 macrophage culture supernatant in the presence and absence of LPS (2 μg/ml, from *Escherichia coli*, serotype O111:B4, Sigma-Aldrich®, Saint Louis, United States of America). In 24-well microplates, the RAW 264.7 cells (3 × 10^5^ cell/well) were cultivated in Dulbecco's Modified Eagle Medium (DMEM) supplemented with 10% fetal bovine serum (FBS) for 24 h at 37°C at an atmosphere of 5% CO_2_. After this period, the medium was aspirated and TanP was added at different concentrations (2–25 μM) in the absence or presence of LPS (2 μg/ml). The plate was maintained under the previously described conditions for 24 h, and the culture medium was collected for cytokines level quantitation in the supernatant. The cytokines were quantified by ELISA using the eBioscience® kit (San Diego, United States of America), following the methodology described by the manufacturer. The results were expressed in picograms per milliliter.

### Cell Viability of 3T3 Cells (ATCC CCL-92)

The MTT assay was employed to assess the viability of 3T3 fibroblast cells (ATCC CCL-92) in the presence of TanP (Mosmann, 1983). The 3T3 cell line (ATCC CCL-92) was kindly provided by Dr. Carmen Ferreira (Department of Biochemistry, UNICAMP, São Paulo, Brazil). In brief, the 3T3 cells were seeded in a 96-well plate (5 × 10^3^ cell/well) and incubated in DMEM with 10% FBS, at 37°C with 5% CO_2_ saturation. After this period, TanP was added at different concentrations (5–50 μM) and incubated for 24 h. Then, the peptide was removed, and 100 μl of MTT (5 mg/ml) in medium was added and further incubated for 4 h at 37°C. The supernatants were removed and replaced by 100 μl of ethanol to solubilize formazan crystals. Measurements were carried out at 570 nm using the microplate reader (Epoch-BioTek^®^). The results were presented as a percentage of MTT reduction, considering the absorbance of the negative control (plate without addition of the peptide) as 100% reduction.

### *In Vitro* Scratch Wound Assay

The 3T3 cells were seeded in a 24-well plate and incubated in DMEM with 10% FBS until cell confluence reached about 80–90%. The cells were further grown for the next 24 h at 37°C in a 5% CO_2_ incubator. A uniform scratch wound was created using a 200-μl sterile pipette tip. To remove loose cells, the wells were washed with PBS (pH, 7.4). The scratched cells were then treated with different concentrations of TanP (2–50 µM). The wells containing only the culture medium were used as the negative control. To monitor the closure of the lesion, images were obtained using the Nikon Eclipse inverted microscope with a 10× objective, at 0, 12, and 24 h, after incubation with TanP. The scratch area was analyzed using the software NIS-Elements AR, considering the results as a percentage of closure of the lesion in relation to the initial area (Balekar et al., 2012; Tonin et al., 2016).

### Statistical Analysis

Statistical analysis was performed using GraphPad Prism software (version 7.0, GraphPad, San Diego, United States of America). All experiments were conducted at least in triplicates. The data analysis was performed using one-way analysis of variance (ANOVA) followed by Tukey's test. The data were expressed as mean ± standard deviation (SD) and considered significant when *p <* 0.05, **p < 0.01, ***p < 0.001, and ****p < 0.0001. For the experimental assay with metals, the program OriginPro 8.5 was used to plot the graphs.

## Results

### Metal-Chelating Properties of TanP

**FIGURE 1:**
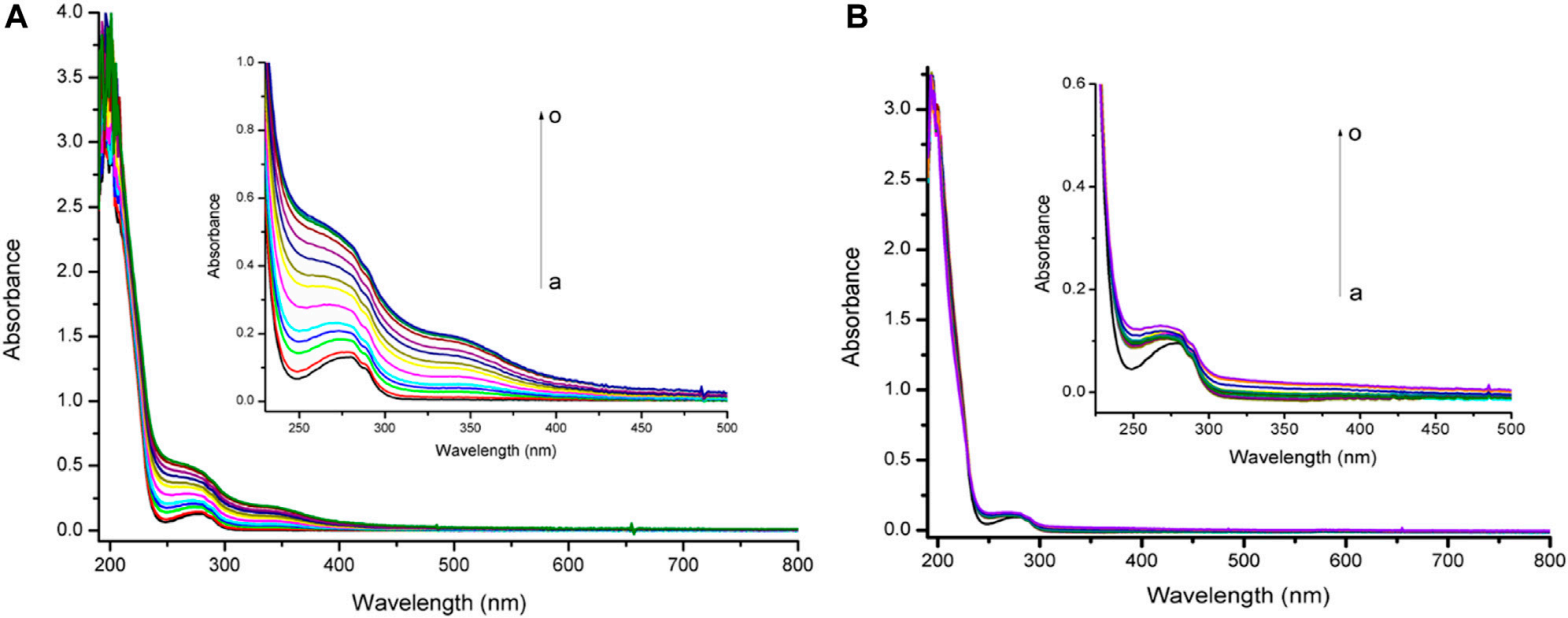
Uv-vis absorption spectra of TanP (25 µM) upon the addition of different concentrations of **(A)** Fe^2+^ and **(B)** Zn^2+^, curves a–o (0–175 μM).

The reactivity assays by UV-visible spectrometry of TanP with different concentrations of metal ions are reported in Figure 1. In the absence of metal ions, the UV-vis spectrum of TanP showed the absorption maximum at 255 and 275 nm (Supplementary Figure S1). When FeSO_4_·7H_2_O was added, changes in the peptide spectral profile in maximum absorption from 255 to 275 nm occurred, indicating the formation of a TanP-Fe^2+^ complex. For Zn^2+^ no relevant spectral change was noted. As shown in Supplementary Figure S2, initially, in the presence of Fe^2+^, the absorbance increased linearly and then became stable, revealing a 1:5 stoichiometry for the complex (TanP: Fe^2+^).

To propose a mode of interaction of TanP with Fe^2+^ and Zn^2+^ ions, the following [Fe(Ac.)_2_(H_2_O)_4_] and [Zn(Ac.)_2_(H_2_O)_2_] complexes were used after conformational searching. The obtained geometries for Zn^2+^ and Fe^2+^ complexes presenting tetrahedral and octahedral shapes, respectively (Supplementary Figure S3). For Zn^2+^, two water molecules and one oxygen of each acetate are located on the vertices of a tetrahedral-like structure. The angles between these four ligands passing by the metallic central atom have an average of 108.7°, which characterizes this geometry, and an average distance of 2.0 Å. As on the Zn^2+^ complexes, dication iron also interacts with one oxygen of each acetate carboxylate; however, four water molecules are present on the first hydration shell and form an octahedral complex. The average angles between the adjacent and opposite oxygen atoms were 90.0° and 174.5°, respectively, over an average distance of 2.0 Å from the central atom.

Theoretical absorption spectra for the two [Fe(Ac.)_2_(H_2_O)_4_] and [Zn(Ac.)_2_(H_2_O)_2_] complexes are displayed in Supplementary Figure S4. These spectra were simulated for the spectral range of wavelengths between 170 and 300 nm, which is consistent with the spectral window used to obtain the experimental data (Supplementary Figure S1). All vertical transitions calculated for the two complexes are characterized (wavelength, oscillator strength, and types of orbitals involved in the transitions) and listed in the Supplementary Table S1. As can be observed in Supplementary Figure S4 and Supplementary Table S1, while the [Zn(Ac.)_2_(H_2_O)_2_] complex presents nine excited states (between 172 and 212 nm), the [Fe(Ac.)_2_(H_2_O)_4_] complex has 20 excited states (between 171 and 255 nm). The NTOs of the main excited state for the [Fe(Ac.)_2_(H_2_O)_4_] and [Zn(Ac.)_2_(H_2_O)_2_] complexes are displayed in Supplementary Figure S4.

On the one hand, these nine excited states of the [Zn (Ac.)_2_(H_2_O)_2_] complex are described mainly from transitions of the highest occupied molecular orbitals (HOMO, HOMO-1, and HOMO-2) centered on the carboxylate ligand (orbitals type *n*) to the lowest unoccupied molecular orbitals (LUMO, LUMO+1, and LUMO+2) also associated with the acetate ligand moiety (type π* orbitals). On the other hand, for the [Fe (Ac.)_2_(H_2_O)_4_] complex, the most important excited states describe a low-intensity band (between 185 and 220 nm), which results mainly from almost isoenergetic transitions originating from the 3d orbitals (HOMO, HOMO-1, and HOMO-2) located at the metallic center to the type π* orbitals located at the acetate ligand (LUMO+3 and LUMO+4).

**FIGURE 2:**
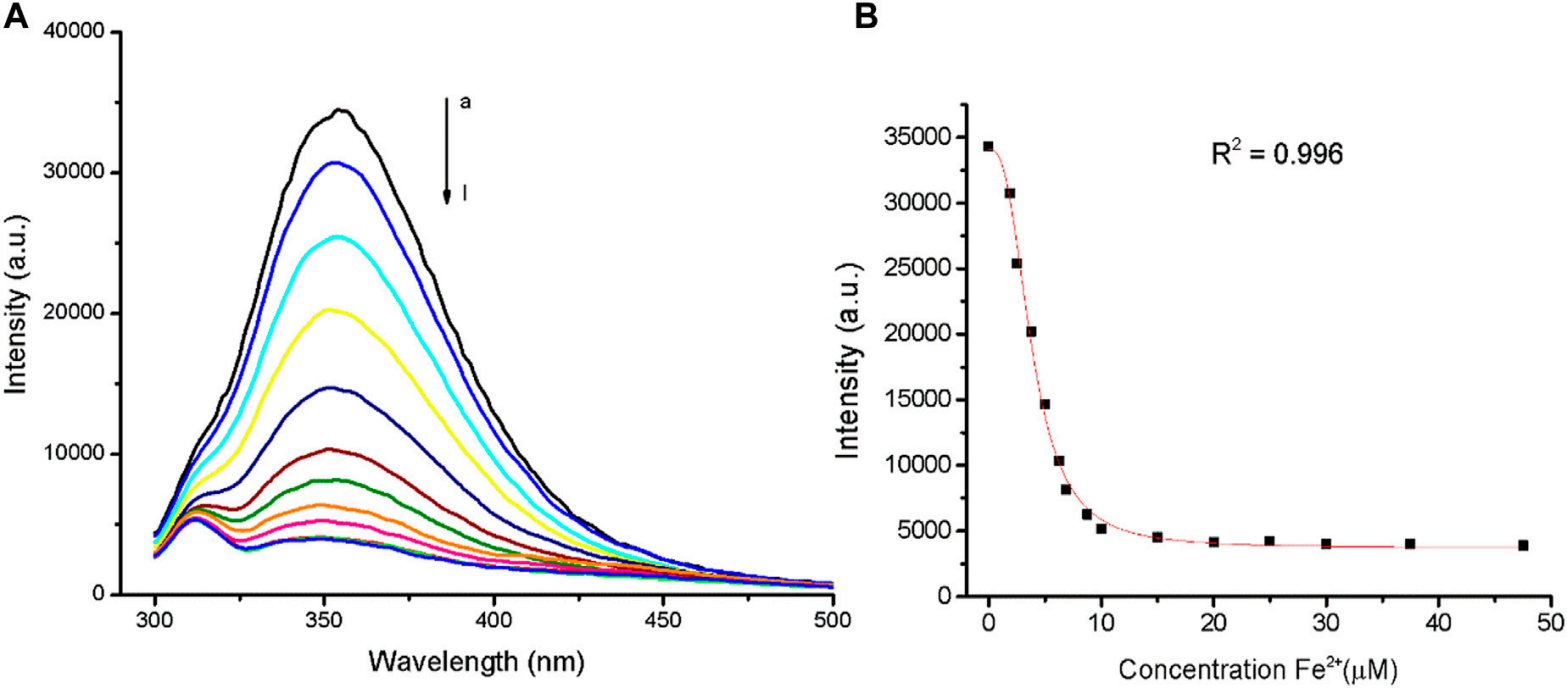
Quenching effects of Fe^2+^ on TanP (1.12 μM) fluorescence intensity, with λ_ex_ = 280 nm. **(A)** Addition of iron ion, curves a–l (0–47.7 μM). **(B)** Fluorescence intensity of TanP at 353 nm with increased Fe^2+^.

In fluorescence emission spectra, TanP (1.12 µM) revealed an intense emission band with λ_max_ around 353 nm when excited at 285 nm, which is in agreement with the tryptophan (Trp) emission range (Supplementary Figure S5). The fluorescence intensity of TanP (1.12 µM) decreased after Fe^2+^ addition (Figure 2A). The highest ratio of 1:7.4 (TanP: Fe^2+^) led to the lowest fluorescence intensity, according to the value obtained in the graph regarding the spectral variations of the emission band as a function of the concentration of the metal ion (Figure 2B). No change in fluorescence emission of TanP was noted by the addition of Zn^2+^ (Supplementary Figure S6).

### Hemolytic Activity

**FIGURE 3:**
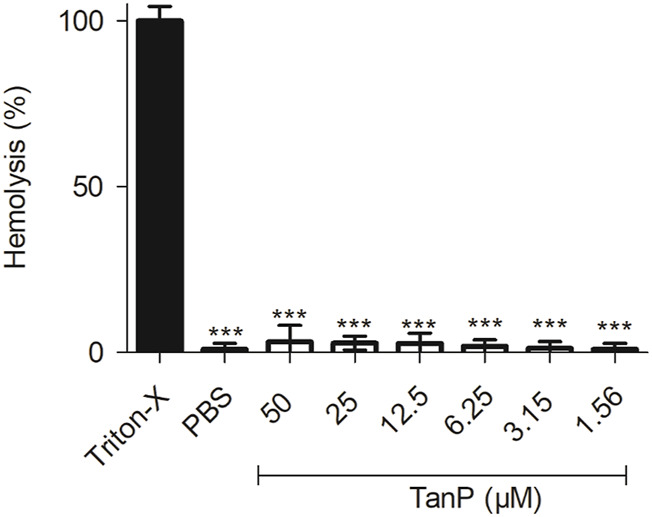
Hemolytic activity of TanP *in vitro*. TanP was tested (1.56–50 μM), the statistical significance was performed using ANOVA followed by Tukey's test and expressed as mean ± SD (n = 3). ***p < 0.001 compared with the positive control group (Triton-X).

TanP showed low hemolytic activity for all concentrations tested (1.56–50 μM), with 3% of hemolytic activity when evaluated at the highest concentration (50 µM), evidencing that the peptide did not cause significant hemolysis effect in red blood cells *in vitro* (Figure 3).

### *In Vitro* Antioxidant Potential of TanP

**FIGURE 4:**
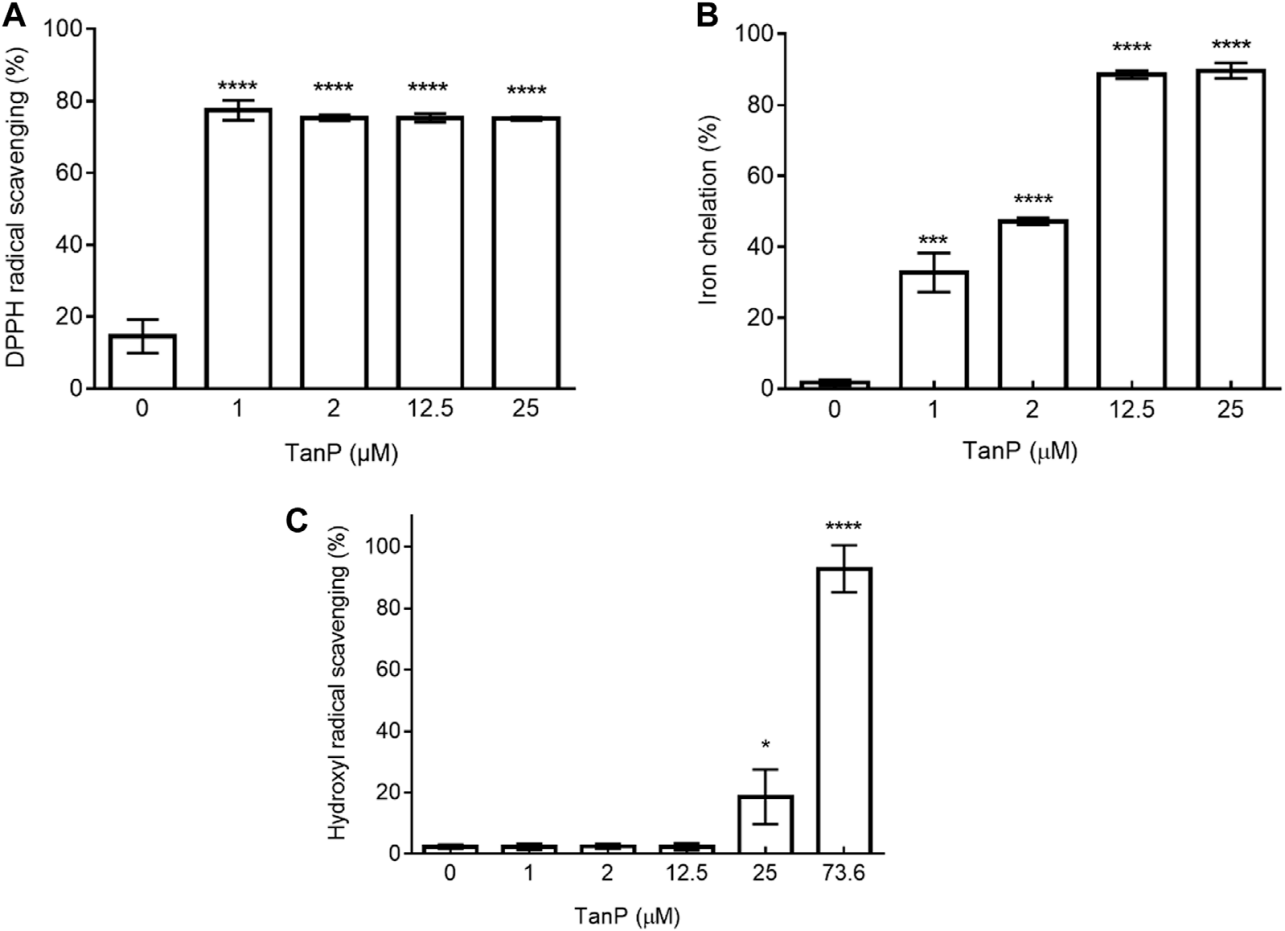
Evaluation of antioxidant activity of TanP. **(A)** DPPH radical scavenging capacity. DPPH solution (150 µM) was used as a control. **(B)** Iron chelation capacity. EDTA (0.1 mg/ml) was used as a control. **(C)** Hydroxyl radical scavenging activity of TanP. Gallic acid (0.25–2 mg/ml) was used as the control. Statistical significance was performed using ANOVA followed by Tukey's test and expressed as mean ± SD (n = 3). *p < 0.05, **p < 0.01, ***p < 0.001, and ****p < 0.0001 compared with the control group.

TanP showed low activity (less 5%) in three different tests, reducing power, superoxide radical–scavenging, and copper-chelating test (data not shown). On the other hand, TanP exhibited DPPH radical–scavenging activity above 70% at all concentrations (1–25 μM) (Figure 4A). For the iron ion chelation test, TanP showed 89.7% activity (Figure 4B) when evaluated at the highest concentration (25 μM). In addition, TanP (73.6 μM) displayed 96% of the hydroxyl radical–scavenging activity (Figure 4C).

### Evaluation Anticoagulant Activity *In Vitro*

**FIGURE 5:**
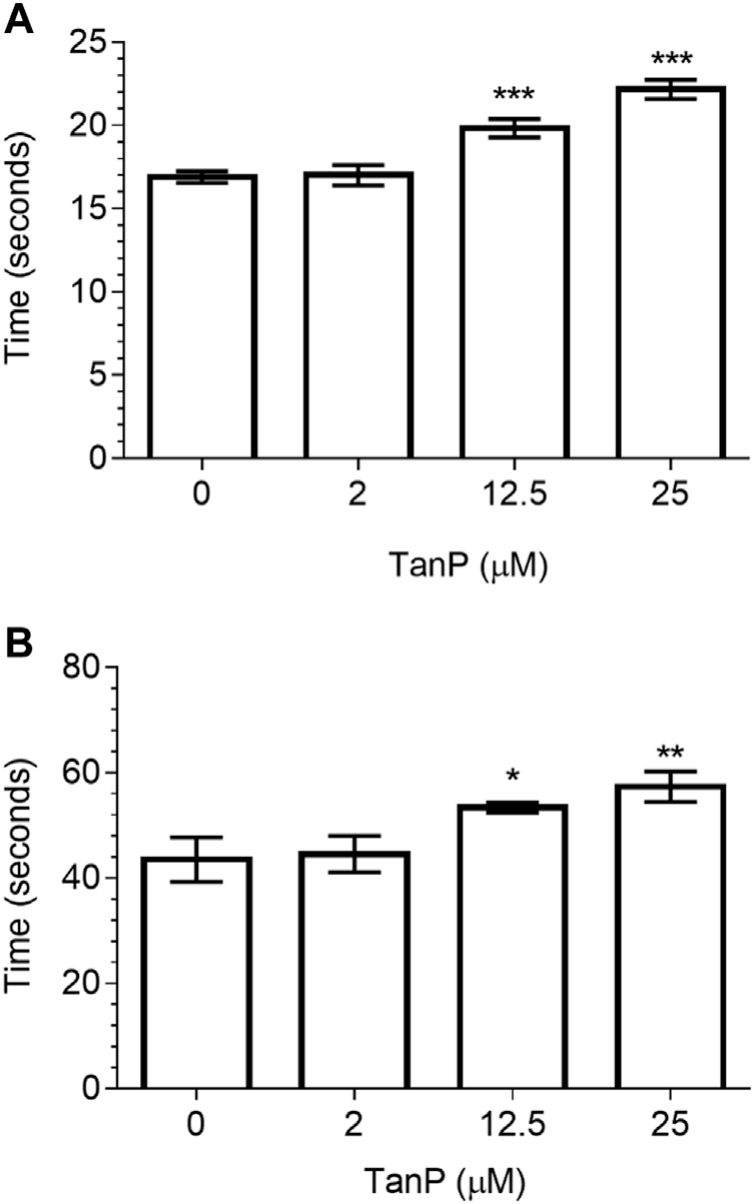
Evaluation of TanP coagulant activity *in vitro*. **(A)** PT and **(B)** aPTT assays. Statistical significance was performed using ANOVA followed by Tukey's test and expressed as mean ± SD (n = 3). *p < 0.05, **p < 0.01, and ***p < 0.001, compared with the negative control group (plasma without TanP).

The anticoagulant activity of TanP was evaluated by the PT and aPTT assays, using normal citrated human plasma. In both assays, TanP revealed the anticoagulant effect at concentrations of 12.5 and 25 µM (Figure 5). In the TP assay, a clotting time of 20 and 22.5 s was found for concentrations of 12.5 and 25 μM, respectively (Figure 5A). In the aPTT assay, a clotting time of 53.4 and 58.4 s was found, for concentrations of 12.5 and 25 μM, respectively (Figure 5B). Plasma with heparin was used as the positive control and as expected presented significant anticoagulant activity, with PT higher than 60 s (seconds of the negative control: 16.27 ± 0.32) and aPTT higher than 240 s (seconds of the negative control: 35.07 ± 0.03).

In addition to the anticoagulant activity, TanP was also tested in relation to its capacity to hydrolyze fibrin and fibrinogen, in view of investigating its potentiality as a thrombolytic agent. At the concentrations evaluated, TanP did not demonstrate fibrinogenolytic activity (data not shown).

### Effect of TanP on the Release of Cytokines

**FIGURE 6:**
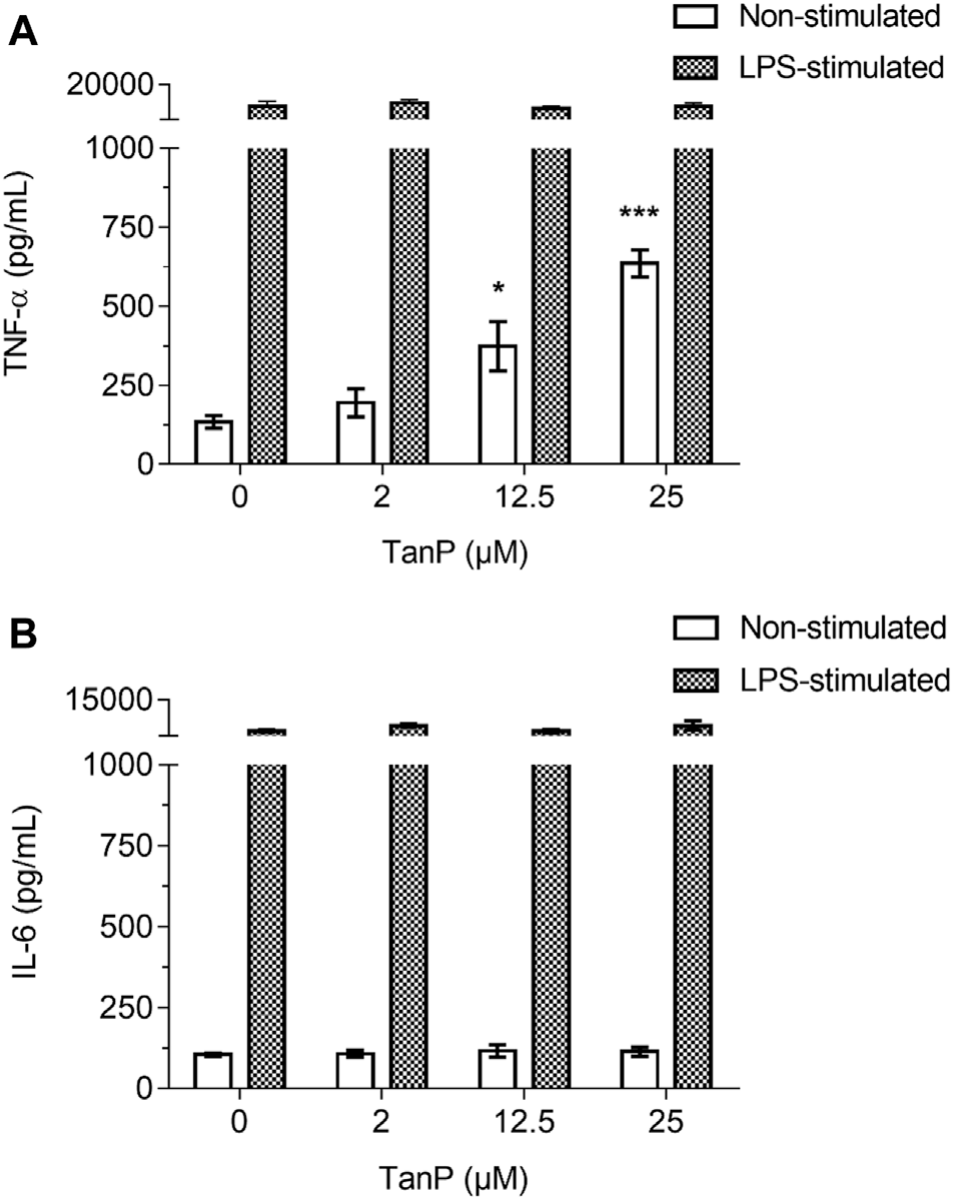
Effect of TanP on pro-inflammatory cytokines levels in murine macrophage (RAW 264.7) supernatant. The release of cytokines **(A)** TNF-α and **(B)** IL-6 in the presence or absence of LPS. The levels of cytokines secretion by cells were measured for 24 h after the interaction with LPS (2 μg/ml) and/or at different concentrations of TanP (0–25 µM). Statistical significance was performed using ANOVA followed by Tukey's test and expressed as mean ± SD (n = 3). *p < 0.05 and ***p < 0.001, compared with the negative control group (cells without TanP or LPS) or the positive control group (cells only with LPS).

TanP (2–25 µM) did not reduce the viability of RAW 264.7 cells when incubated for 24 h, indicating a nontoxic character for this cell line (Supplementary Figure S7). The peptide induced a distinct release profile of the pro-inflammatory cytokines TNF-α and IL-6 in the absence of LPS. An increase in TNF-α expression of 316.26 and 622.56 pg/ml was observed in the supernatant of the murine macrophage culture in the presence of 12.5 and 25 µM of TanP, respectively, revealing a concentration-dependent relationship (Figure 6A). No significant changes were observed for IL-6 levels (Figure 6B). In the presence of LPS, TanP (2–25 µM) did not induce changes in the release profile of the TNF-α and IL-6 cytokines when incubated for 24 h.

### Cell Viability of 3T3 Cells

TanP (2–50 µM) neither induced the proliferation of 3T3 cells nor reduced their viability when incubated for 24 h, indicating a nontoxic character for this cell line (Supplementary Figure S8).

### Evaluation of the Healing Potential of TanP Using Cell Migration Assay

**FIGURE 7:**
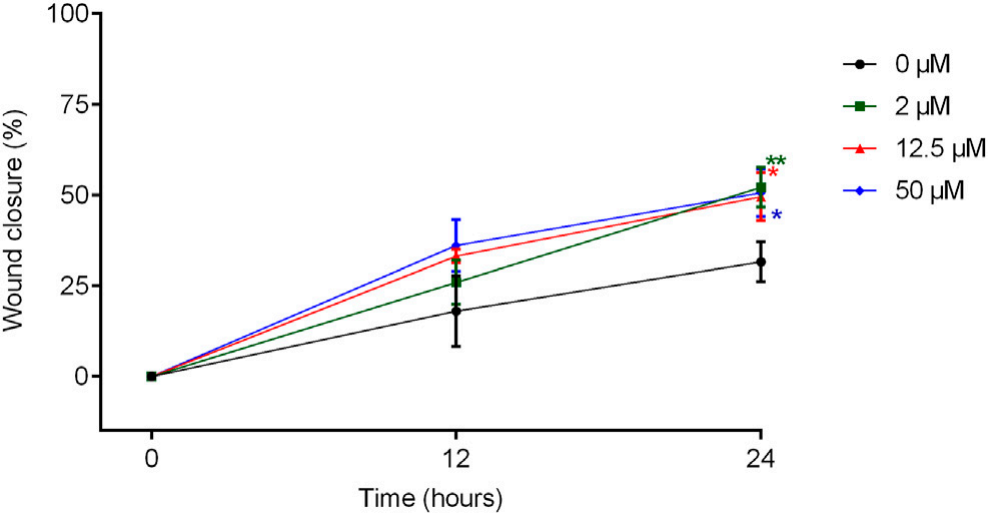
Cell migration of 3T3 cells treated with TanP after the Scratch assay. Fibroblast confluence after scratching and treatment with different concentrations of TanP (0–50 µM) was analyzed at 0, 12, and 24 h. The areas were measured with the aid of the software NIS-Elements AR. Statistical significance was performed using ANOVA followed by Tukey's test and expressed as mean ± SD (n = 3). *p < 0.05 and **p < 0.01, compared with the control group (without TanP).

Figure 7 shows the effect of treatment with TanP at different concentrations (2–50 µM), at times 0, 12, and 24 h, with respect to the migration of fibroblasts, using the scratch method.

When the measurements of the scratch areas were compared, a significant increase in the percentage of lesion closure was observed after 24 h of incubation with the peptide, for all concentrations evaluated in relation to the group control. In addition, in the presence of TanP (2 μM, 12.5 and 50 µM), approximately 50% of the wound was closed after 24 h of treatment, whereas for the control group, this closure was only 30%.

## Discussion

High concentrations of Fe^2+^ and Cu^2+^ ions cause these metals to react with molecular oxygen, generating reactive oxygen species (ROS) that damage DNA, lipids, and proteins (Di Bella et al., 2017). Furthermore, cancer, diabetes, arteriosclerosis, inflammatory disease, autoimmunity, cardiovascular disease, and Alzheimer's disease have been associated with the increase of ROS or the inability of the organism to reduce these reactive species (Nascimento et al., 2013). The development of multifunctional molecules, including natural products, capable of simultaneously combating several pathological characteristics, acting as chelating agents, antioxidants, anti-inflammatories, and peptide aggregation reducers, among others, is considered a new perspective for the treatment of neurodegenerative diseases (Sales et al., 2019). In this context, components present in the scorpion venom have demonstrated anti-inflammatory (Veloso Júnior et al., 2019), chelating (Melo et al., 2017), and antioxidant action (Daniele-Silva et al., 2021), constituting a promising source for the development of new drugs.

In this study, TanP revealed a significant affinity on iron (II), a biologically important metal ion, not showing a similar affinity profile with respect to the zinc (II) metal, in the experimental conditions observed (Figure 1).

Complexation with Fe^2+^ ions is commonly related to obtaining a compound of octahedral geometry. Coordination sites involving sulfur, nitrogen, and oxygen donors promote this interaction (Bal et al., 2013; Antonietti et al., 2017). In the literature, it is suggested that the metal-binding sites in iron-chelating peptides may also be the carboxyl groups of the aspartic and glutamic residues (Lv et al., 2009; Caetano-Silva et al., 2018), terminal amino and carboxylate groups, and the peptide bonds of the peptide structure, as well as the amino and arginine imine, lysine amino, and histidine imine. In addition, it was found that the absence of these amino acid residues in the peptide chain results in less iron-chelating potential (Wu et al., 2017). Thus, it is suggested that Fe^2+^ ions form a complex with TanP possibly by binding to the oxygen atoms of the carboxylate groups of the aspartic and glutamic acid side chains.

The tetrahedral geometry obtained for the [Zn(Ac.)_2_(H_2_O)_2_] complex in the computational assay of this study revealed a configuration commonly obtained for dication zinc complexes, mainly bio-complexes since the zinc ion interacts with donors such as oxygen and nitrogen of enzyme side chains to generate stronger complexes (Krezel and Maret, 2016). Regarding the geometry obtained for the [Fe(Ac.)_2_(H_2_O)_4_] complex, this is in accordance with several iron complexes that generate the octahedral shape on an aqueous medium, including biological molecules as the heme group (Poulos, 2014).

Our NBO analyses (Supplementary Figure S9, S10, and S11) indicate that while for the [Fe(Ac.)_2_(H_2_O)_4_] complex, the 3d orbitals of the metallic center are set near the HOMO–LUMO frontier orbitals, for the [Zn(Ac.)_2_(H_2_O)_2_] complex, the 3d orbitals of zinc are far from the boundary orbitals. Then, considering the participation of the relevant molecular orbitals, for the [Zn(Ac.)_2_(H_2_O)_2_] complex, the excited states can be assigned mainly as transitions involving just orbitals of aspartate ligand. Differently, for the [Fe(Ac.)_2_(H_2_O)_4_] complex, the main absorption band can be assigned mainly as a metal-to-ligand charge transfer. These theoretical results suggest that by UV-vis spectroscopy: 1) TanP can work as a sensor to identify and quantify (at the evaluated concentrations) iron (II) ions; and 2) TanP does not seem to have sensibility for bivalent zinc (at the evaluated concentrations and spectral range considered).

Conformational changes of proteins can be monitored using fluorescent probes or intrinsic fluorescence, which is caused by aromatic amino acid residues (tryptophan, tyrosine, and phenylalanine) (Zhdanova et al., 2015). In the primary sequence, TanP contains one tyrosine residue, three phenylalanine residues, and one tryptophan residue, which contribute to the intrinsic fluorescence of the peptide.

When evaluated by fluorescence spectroscopy, TanP displayed an intense emission band with λ_max_ around 353 nm, when excited at 285 nm (Supplementary Figure S5), which corroborates with the maximum emission length of tryptophan, which varies from 310 to 350 nm, depending on the electrostatic environment (Adams et al., 2002). For the other chromophores, tyrosine and phenylalanine amino acids, the emission spectrum is in the 290 nm range, which overlaps with the absorption spectrum of tryptophan, causing an energy transfer from these amino acids to tryptophan, making this the dominant chromophore in the fluorescence process of peptides and proteins (Zhdanova et al., 2015).

The changes observed in TanP emission bands, in the presence of Fe^2+^, corroborate with the results obtained in UV-vis spectroscopy. The lower concentration of TanP (1.12 μM) coordinates more iron ions, 1:7.4 (TanP:Fe^2+^). A similar proportion (TanP:metal) was reported in the UV-vis copper study, for a peptide concentration of 2.11 μM, mentioning a proportion of 1:7 (TanP:Cu^2+^) (Melo et al., 2017). In addition, it can be suggested that the metal is binding to a site close to the amino acid tryptophan.

The presence of molecules with antioxidant effects in scorpions has been reported in the literature (Wali et al., 2020). The Stigmurin (FFSLIPSLVGGLISAFK-NH2), cationic peptide of *T. stigmurus*, showed hydroxyl radical scavenging above 70% at 10 μM (Daniele-Silva et al., 2021). A peptide fraction isolated from the venom of *Buthus occitanus* has been demonstrated to exhibit the antioxidant and free radical scavenger effects (Bekheet et al., 2013). Antioxidant peptides from *B. martensii* Karsch were separated and purified, and showed the highest ABTS^+^-scavenging activity and the highest DPPH-scavenging activity, but the OH-scavenging activities of these peptides were not significant (Wali et al., 2020).

The substrate oxidation process consists of three stages (initiation, propagation, and termination). Antioxidants can act at any of these steps, and the more steps a compound intervenes at, the better the antioxidant it is (Wali et al., 2020). Several *in vitro* antioxidant tests are available to assess the antioxidant activity of biomolecules (Gulcin, 2020). In this current study, we used six methods to evaluate the possible effect of TanP on the initiation (DPPH, iron, and copper chelation), propagation (reducing power), and termination (superoxide and hydroxyl radical–scavenging activities) steps.

In this approach, TanP revealed significant iron-chelating activity, reaching up to above 90% of chelation (Figure 4B), being a pioneer in demonstrating the antioxidant potential of anionic peptides present in the scorpion venom. Chelation of metal iron has an antioxidant effect because the transition metal iron, just like copper, catalyzes the generation of ROS, including hydroxyl radical and superoxide radical, leading to the oxidation of unsaturated lipids and promoting oxidative damage at different levels (Nascimento et al., 2013).

Among ROS, the hydroxyl radical is the most reactive in chemistry. It can abstract hydrogen atoms from biological thiol molecules and form sulfur radicals capable of combining with oxygen to generate oxysulfur radicals and damage biological molecules (Singh and Singh, 2008; Melo-Silveira et al., 2012).

Overall, TanP revealed an antioxidant effect in two different stages of substrate oxidation process; initiation (DPPH reduction and ion chelation) and termination (hydroxyl radical scavenging) (Figure 4).

Different amino acid residues may be responsible for the antioxidant activity in peptides, which is usually due to chelation of transition metals and scavenging of free radicals (Carrasco-Castilla et al., 2012). The high content of hydrophobic amino acids in peptides was mainly responsible for the antioxidant activity (Wali et al., 2020). In addition, nucleophilic sulfur-containing side chains in cysteine and methionine residues, and aromatic side chains in tryptophan, tyrosine, and phenylalanine residues can easily donate hydrogen atoms (Carrasco-Castilla et al., 2012). In its composition, TanP has 23 hydrophobic residues, including one tyrosine, one tryptophan, and four phenylalanine.

Many animal venoms have shown the ability to act on the human hemostatic system as procoagulant or anticoagulant agents (Brazón et al., 2009). In this study, TanP was able to prolong the clotting time in PT and aPTT tests in the highest concentrations (Figure 5), demonstrating anticoagulant activity.

Some scorpion venoms cause blood clotting disorders, but the number of coagulopathic compounds studied to date is quite less (Félix-Silva et al., 2014). Dipeptides isolated from *Heterometrus laoticus* scorpion venom showed no anticoagulant activity at concentrations up to 100 µM in PT and aPTT tests with the human plasma, but they strongly prolonged the bleeding time from mouse tail and in *in vitro* clot formation, through the inhibition of platelet aggregation (Tran et al., 2017).

Discreplasminin, a peptide isolated from *Tityus discrepans* venom, showed antifibrinolytic activity, since it inhibits plasmin; although, a previous study had shown the prolongation of human plasma PT and aPTT in the presence of the venom (Brazón et al., 2009). The hydrolyzate from the BmK protein of scorpion (*B. martensii* Karsch) exhibited high anticoagulant activity, and this action has been associated with the presence of negatively charged amino acids and hydrophobic residues (Ren et al., 2014).

Additionally, TanP did not demonstrate the ability to hydrolyze fibrinogen in tested concentrations. Differently from the results obtained in this study, it was identified in *T. discrepans* that fibrinogenolytic enzymes are responsible for the degradation of the fibrinogen Aα and Bβ chains, and these mechanisms were also related to the prolongation of TP and activated aPTT produced by the venom of *T. discrepans* (Brazón et al., 2014), demonstrating that components present in scorpion venom can induce a wide variability of effects on hemostasis.

Our results suggest that the observed anticoagulant activity may be due to an inhibitory action upon clotting factors. Another hypothesis for the observed anticoagulant action is that the possible target of peptide intervention may be platelet aggregation, since peptides isolated from scorpions have this mechanism already elucidated (Thien et al., 2017).

Although other scorpion venom peptides with anticoagulant activity are described in the literature, this is the first study that demonstrates that anionic peptides present in scorpion venom have this activity. However, given the results that have been presented, further studies are necessary to elucidate the exact mechanism of the anticoagulant effects of TanP.

Venoms and toxins are responsible for modulating the immune response (Petricevich, 2002; Petricevich and Lebrun, 2005). Envenoming by different species of scorpion, even the *Tityus* genus, results in the release of pro- and anti-inflammatory cytokines, and the balance between such cytokines in the poisoning determines the degree and extent of inflammation, which can lead to important clinical effects, such as cardiac dysfunction, pulmonary edema, and shock (Petricevich, 2010).

The macrophages are cells that participate in all stages of the inflammatory process, since phagocytosis helps in the production of chemokines, cytokines, and growth factors. In addition, the macrophages present extensive phenotypic and functional plasticity, whose regulation critically defines beneficial or detrimental outcomes in inflammatory responses (Mendes et al., 2019). TanP promoted the release of TNF-α, in murine macrophages, in the absence of LPS (Figure 6A). It is not known whether stimulation of TNF-α cytokine production arises from a nonspecific interaction of the peptide with the macrophage membrane or from interaction with a specific receptor. However, TanP is unlikely to bind to the LPS receptor since the effects of LPS on cytokine production were not affected in the presence of the peptide.

Previous studies with *T. serrulatus* scorpion venom (TSV) demonstrated that the incubation of macrophages with TSV provided an increase in the production of IL-6 and IFN- γ, but there was no detection of TNF-α in the cell supernatant (Petricevich, 2002). However, when evaluating the activity of fractions isolated from this venom, it was found that the FII fraction is a potent activator of the production of macrophage TNF-α (Petricevich and Lebrun, 2005).

Basic amino acids present in the peptide chain are important residues for interaction with the target in macrophages. Cationic peptides isolated from *T. serrulatus* venom were able to modulate macrophage responses, increasing the release of IL-6 (Pucca et al., 2016). ToAP3 and ToAP4, cationic peptides obtained from *T. obscurus* venom, have been demonstrated to be a potential *in vitro* immunomodulator on murine bone marrow–derived macrophages stimulated by LPS, being able to reduce the release of TNF-α. This stimulation is associated with peptide interaction with toll-like receptor 4 (TLR4). However, no increase in cytokine levels was observed when both cells were treated with ToAP3 or ToAP4 alone (Veloso Júnior et al., 2019). Although TanP had no basic amino acid residues in its composition and was rich in acidic and hydrophobic residues, it was able to modulate cytokine release by macrophages in the absence of LPS. In murine macrophages, the LPS binds with a carrier of LPS-binding protein (LBP). The LPS-LBP complex interacts with some receptors such as CD14, MD2, and TLR-4 proteins that trigger the activation of an intracellular cascade that induces the activation of the transcription factor nuclear factor kappa B to the nucleus, which is responsible for the transcription of pro-inflammatory genes, resulting in the production of inflammatory cytokines and the expression of co-stimulatory molecules (Torres-Rêgo et al., 2016; Zamyatina and Heine, 2020). The mechanism involved in the immunomodulatory effect by TanP is still unclear, and more tests should be carried out for elucidation. However, it is possible that TanP interacts with TLR4, inducing the production of TNF-α. This interaction is significantly less than the power of the receptor to recognize LPS, so the presence of the peptide does not interfere with the activation caused by LPS.

The release of nitrite by RAW macrophages in the presence of LPS was inhibited by TanP, indicating that the peptide neutralizes LPS-induced nitric oxide production. TanP treatment without LPS stimulation reached the same level as the negative control (Melo et al., 2017). Thus, it is suggested that TanP has an immunomodulatory potential because in unstimulated macrophages, it can increase the release of inflammatory mediators, while in the presence of LPS, it decreases the production of nitric oxide, thus preventing exacerbated inflammatory reactions. There are a few studies involving the immunomodulatory potential of anionic peptides. To date, no studies with anionic peptide from scorpion venom in the approach of the immunomodulatory activity are known.

Considering a skin lesion, wound healing after hemostasis occurs in three overlapping stages: inflammation, proliferation, and remodeling. Fibroblasts are critical in all the three phases (Desjardins-Park et al., 2018). Fibroblast migration can accelerate the wound's revitalization process and promote its closure during healing (Liu et al., 2014; Yang et al., 2019).

In this study, it was demonstrated that TanP (2–50 µM) does not exhibit the potential to promote the proliferation of 3T3 cells *in vitro*, but a significant increase in the percentage of lesion closure was observed after 24 h of incubation with the peptide in certain concentrations, due to the action on fibroblast migration (Figure 7). Thus, it is suggested that TanP may be acting as an exogenous fibroblast growth factor, or it may potentiate the activity of existing fibroblast growth factors.

Chronic wounds contain high levels of reactive oxygen and nitrogen species. The overproduction of free radicals, together with the accumulation of iron ions, perpetuates the inflammatory phase, resulting in severe tissue damage. For this reason, the introduction of antioxidants seems to be a promising strategy to promote normal wound healing (Pivec et al., 2019).

Iron-chelating molecules, such as deferoxamine, have high wound healing potential, even when dealing with diabetic patients, suggesting that iron depletion is beneficial in endothelial dysfunction in diabetes (Duscher et al., 2018). Thus, as TanP was able to chelate iron ions, there is a perspective that this function may contribute to the wound healing process.

In the inflammation process, at the initial healing stage, excessive inflammatory mediators, such as radicals, are released at the wound site, often associated with oxidative stress and subsequent prolonged inflammation, resulting in difficulty in healing the wound (Zhao et al., 2019). Therefore, since TanP also showed significant results with respect to chelating and antioxidant properties, and anti-inflammatory capacity, this peptide presents a high potential to be applied as a prototype to obtain new healing agents.

This is the first study to approach the role of anionic peptides of scorpions in immunomodulation and the wound healing process. Furthermore, TanP displayed the ability to chelate Fe^2+^ ions and revealed antioxidant and anticoagulant potential. This approach provides preliminary results regarding the therapeutic potential of TanP, which serve as a basis for the development of new studies in search of a prototype of a new drug. Besides, TanP has a potential for biotechnological application and can be used as a biosensor for identifying and quantifying Fe^2+^ ions.

## Data Availability

The data sets presented in this study can be found in online repositories. The names of the repository/repositories and accession number(s) can be found in the article/Supplementary Material.
